# Activated human B cells produce phospholipase D4-containing extracellular vesicles

**DOI:** 10.1371/journal.pone.0329832

**Published:** 2025-08-14

**Authors:** Tsubasa Betsuyaku, Shuji Akizuki, Yihan Liu, Akio Morinobu

**Affiliations:** Department of Rheumatology and Clinical Immunology, Graduate school of Medicine, Kyoto University, Kyoto, Japan; Nathan S Kline Institute, UNITED STATES OF AMERICA

## Abstract

Phospholipase D4 (PLD4) is an intracellular exonuclease implicated in immune regulation via nucleic acid degradation. While public proteomic databases indicate the presence of PLD4 in human plasma, its mode of extracellular release remains unclear. This study demonstrates that activated B cells secrete PLD4-containing extracellular vesicles (EVs), providing a novel mechanism for its extracellular presence. EVs were purified from human plasma and confirmed to contain PLD4 through immunoelectron microscopy (IEM), Western blotting, and enzyme-linked immunosorbent assay. To further investigate the cellular origin, human B cells were stimulated in vitro with B cell receptor engagement and TLR9 agonist. Light and electron microscopy revealed significant cellular hypertrophy and accumulation of multivesicular bodies following stimulation. Nanoparticle tracking analysis (NTA) confirmed an increase in EV secretion, and IEM demonstrated a higher frequency of PLD4-positive EVs in stimulated B cells. Additionally, immunofluorescence and IEM revealed that PLD4 relocates from the Golgi apparatus to CD63-positive endosomes, where it is incorporated into intraluminal vesicles prior to EV release. These findings establish that activated B cells contribute to the extracellular distribution of PLD4 via EV secretion, highlighting a potential role in intercellular communication and immune regulation.

## Introduction

Endogenous nucleases, such as DNase and RNase, play a crucial role in maintaining biological homeostasis by cleaving both self-derived and foreign nucleic acids. For each nuclease to function properly, it is essential that it is localized correctly within specific intra- and extracellular compartments [1].

Phospholipase D3 (PLD3) and Phospholipase D4 (PLD4) are recently identified intracellular exonucleases with two phosphodiesterase (PDE) active sites, capable of digesting single-stranded DNA (ssDNA) and single-stranded RNA (ssRNA) under acidic pH conditions [2]. Unlike classical members of the phospholipase D family, such as PLD1 and PLD2, both PLD3 and PLD4 have an N-terminal transmembrane domain that directs their localization to intracellular organelles [3]. Due to their distribution and enzymatic activity in acidic environments, PLD3 and PLD4 primarily degrade nucleic acids in endosomes and lysosomes, resulting in a reduction of Toll-like receptor (TLR7 and TLR9) ligands and thereby negatively regulating the immune responses triggered by these pattern recognition receptors [4].

At the organismal level, PLD4 has been reported to be upregulated in activated microglia, where it contributes to phagocytic function and is involved in cerebellar development in neonatal mice [5] as well as the suppression of renal fibrosis in renal tubules [6]. Genetic studies have linked single nucleotide polymorphisms (SNPs) in PLD4 to the onset of rheumatoid arthritis [7], systemic lupus erythematosus (SLE) [8], and systemic sclerosis [9]. Additionally, PLD3 and PLD4 knockout mice exhibit systemic inflammation characterized by excessive interferon-γ (IFNγ) production and liver damage [10]. Mice with a nonsense mutation in PLD4 develop SLE-like autoantibody production and nephritis in a TLR9-dependent manner [8], highlighting the critical role of PLD4 in maintaining immune tolerance across species.

In contrast, several nucleases have been reported to exhibit non-canonical molecular distribution within the organism. For instance, DNASE1L3, an extracellular nuclease, possesses an N-terminal secretory signal sequence and is typically secreted to degrade excess extracellular nucleic acids. However, DNASE1L3 has also been shown to inhibit the release of nuclear proteins during apoptosis and suppress the nuclear release of related proteins during inflammasome activation [11,12]. Interestingly, despite their differing typical localizations—DNASE1L3 being extracellular and PLD4 intracellular—both DNASE1L3 and PLD4 are associated with human autoimmune diseases [13,14] and display similar phenotypes in genetically modified mice [12]. Moreover, data from public databases indicate that these molecules are expressed in overlapping human immune cell types, including plasmacytoid dendritic cells, B cells, and monocytes [15,16].

In addition to these findings, protein mass spectrometry data from human plasma suggest that PLD4 is present extracellularly and is more abundant than the more common extracellular nuclease DNASE1 [15,16]. This observation leads to the hypothesis that PLD4 may have physiological functions beyond its role as an intracellular nuclease, potentially extending to the extracellular space.

Considering this background, the aim of this study is to investigate the extracellular presence of PLD4. Specifically, given reports suggesting that PLD4 functions as a membrane protein, we hypothesize that PLD4 may be incorporated into extracellular vesicles and released into the extracellular environment.

## Materials and methods

### Study approval

All experimental protocols were approved by the Animal Care and Use Committee of the Kyoto University Institute. All experiments were conducted in accordance with the institutional guidelines and the principles of the Helsinki Declaration of 1975 (revised in 2008) [8].

### Antibodies and kits

The following antibodies were used: Recombinant human anti-human PLD4 therapeutic antibody biotinylated (TAB-759CL-Biotin; Creative Biolabs, NY, USA); rabbit anti-PLD4 polyclonal antibody (HPA051512; Atlas Antibodies, Bromma, Sweden); rat anti-CD9 monoclonal antibody (017−28211; FUJIFILM Wako, Osaka, Japan); FITC mouse anti-human CD63 antibody (353005; BioLegend, San Diego, CA, USA); PerCP/Cyanine5.5 mouse anti-human CD19 antibody (302230; BioLegend); Mouse anti-human CD19 antibody (302202; BioLegend); rabbit anti-TGN46 polyclonal antibody (13573–1-AP; Protein Tech, IL, USA).

We utilized the EasySep^TM^ Human Pan-B Cell Enrichment Kit (19554; STEMCELL Technologies, Vancouver, Canada) to isolate pan-B cells from human peripheral blood mononuclear cells (PBMCs). For immunofluorescence microscopy, SlowFade^TM^ Diamond Antifade Mountant with 4’,6-diamidino-2-phenylindole (DAPI) (S36068; Invitrogen, Carlsbad, CA, USA) was used for counterstaining. Plasma membranes were stained using the MemBrite^®^ Fix Cell Surface Staining Kits – MemBrite^®^ Fix 680/700 (SKU: 30099-T; Biotium, Fremont, CA, USA). B cell stimulation was achieved using ODN2006 (tlrl-2006; InvivoGen, San Diego, CA, USA) and/or AffiniPure F(ab′)2 Fragment Goat Anti-Human IgG + IgM (H + L) (109-006-127; Jackson ImmunoResearch Laboratories, Inc., West Grove, PA, USA). Endogenous biotin inactivation was conducted using the Endogenous Biotin Blocking Kit (E21390; Invitrogen).

### *In Vitro* experiment

Five healthy participants were recruited, and blood samples were collected [17]. All study participants provided informed consent. From these blood samples, human PBMCs were isolated by density gradient centrifugation. Subsequently, B cells were isolated using the EasySep^TM^ Human Pan-B Cell Enrichment Kit according to the manufacturer’s protocol [18]. The purified B cells were cultured in RPMI1640 (R8758; Sigma-Aldrich Co. LLC; St. Louis, MO, USA) supplemented with 10% fetal bovine serum (26140−079; Grand Island, NY, USA), L-glutamine (25030; GIBCO), penicillin-streptomycin (15140−122, GIBCO), HEPES (15630; GIBCO), sodium pyruvate (S8636, Sigma-Aldrich), and 2-mercaptoethanol (M-3148; Sigma-Aldrich). Thereafter, cultured B cells were exposed to 0.25nM of CpG and/or 2.5 μg/mL of anti-IgG + M F(ab′)2 for 48 h before analysis [19]. For further analysis, purified and harvested B cells were fixed in 10% formaldehyde neutral buffer solution (37152−51; Nacalai Tesque, Kyoto, Japan) for light microscopy or in 2% paraformaldehyde and 2.5% glutaraldehyde in 0.1 M phosphate for epoxy embedding and transmission electron microscopy (TEM). Purified EVs were frozen at −80°C for negative staining and TEM [20,21].

### PLD4 overexpression in HEK293T cells

HEK293T cells were transfected with pCMV6-human PLD4 (SKU SC319634; OriGene Technologies, Inc., Rockville, MD, USA) using Lipofectamine^®^ 3000 transfection reagent (L3000015; Thermo Fisher Scientific Inc. Waltham, MA, USA). All the procedures were performed under the manufacturer’s protocol.

### Purification of EVs

EVs were purified from either human plasma or the supernatant obtained from human primary B cell cultures. The samples were centrifuged at 300 × *g* for 10 min, 2000 × *g* for 10 min, 10 000 × *g* for 30 min, and then twice ultracentrifuged at 120 000 × *g* for 100 min at 4 °C using an Optima^TM^ L-100K ultracentrifuge (Beckman Coulter, Brea, CA, USA). EV pellets were subsequently resuspended in PBS and stored at −80 °C until further use, in accordance with the Minimal Information for Studies of Extracellular Vesicles 2018 (MISEV2018) guidelines [22].

### Immunofluorescense light microscopy

Deparaffinized 4μm-sections underwent antigen retrieval, blocking, and then incubated with primary antibodies overnight at 4°C. Subsequently, the samples were labeled with either secondary antibodies or streptavidin for 1 h and counterstained with DAPI. The slides were observed using a Keyence BZ-X810 immunofluorescence microscope (Osaka, Japan). The quantification process involved counting CD63- or PLD4-positive areas and CD63/PLD4 double-positive areas in 5 randomly selected fields for each group. Quantitative analysis of each molecule in high-power fields (1320x) was performed using the ImageJ software (http://imagej.nih.gov/ij/) [20].

### TEM and quantitative analysis

Ultrathin sections (70 nm) from blocks embedded in epoxy resin were double-stained with uranyl acetate and lead citrate and observed using an H7650 transmission electron microscope (Hitachi, Japan) [20]. For cell size quantification, B cells were counted from 10 randomly selected lower-magnification fields (3000 ×), along with the calculation of the nucleus-to-cytoplasm (N/C) ratio using the ImageJ software. Additionally, intraluminal vesicles (ILVs) were counted from the same 10 lower-magnification fields (3000 ×). To observe EVs from the B cell culture supernatant, negative staining was applied, and samples were examined using the H-7650 transmission electron microscope.

### Negative staining and immunogold labeling of PLD4 for EVs

Purified EVs, placed on grids coated with Excell support film (Nisshin EM, Tokyo, Japan), were incubated with 1% bovine serum albumin in Tris buffer for 30 min and then with PLD4 antibody solution (1/10 dilution) for 1 h. After washing with Tris buffer, grids were incubated with streptavidin conjugated with 10 nm gold particles for PLD4 single staining or secondary antibody conjugated with 5 nm gold particles for PLD4/CD19 double labeling (1/50 dilution). Sequentially, grids were incubated with CD19 antibody solution (1/10 dilution) for 1 h. After washing with Tris buffer, grids were incubated with secondary antibody conjugated with 20 nm gold particles (1/50 dilution). Subsequently, the EVs were stained using a modified negative staining method [23]. Gold particles present on EVs were counted across more than 10 randomly selected TEM fields (50 000 ×) using an H-7650 transmission electron microscope [24–26].

### Immunogold labeling of PLD4 for ultrathin sections

Ultrathin sections were etched with saturated sodium metaperiodate and hydrochloric acid, followed by washing with filtered distilled water. Subsequently, they were incubated with 0.1% bovine serum albumin in Tris buffer for 30 min and then with PLD4 antibody solution (1/10 dilution) for 1 h. After washing with Tris buffer, ultrathin sections were incubated with secondary antibody or streptavidin conjugated with 10 nm gold particles (1/50 dilution). The grids were washed and counterstained with uranyl acetate for 10 min as previously described [20]. Gold particles located on ILVs were counted from 10 random MVBs using an H-7650 transmission electron microscope [24,25].

### Flow cytometry

Purified B cells were stained with primary antibodies conjugated with fluorescence dyes or 7-aminoactinomycin D (7-AAD) to detect dead cells. The stained cells were analyzed using the LSR^TM^ Fortessa flow cytometer (Becton Dickinson, Franklin Lakes, NJ, USA), and the data were further processed and analyzed using the FlowJo software (Becton Dickinson) [8].

### Western blot analysis

We extracted 8 μg of protein from purified EVs in the supernatants of post-ultracentrifugation plasma and bulk plasma. These protein samples were solubilized in SDS lysis buffer and then electrophoresed and transferred onto polyvinylidene difluoride membranes. Subsequently, the membranes were blocked with 5% bovine serum albumin and incubated with anti-PLD4 and anti-CD63 antibodies. This was followed by incubation with peroxidase-labeled goat anti-rabbit IgG antibody [20]. Quantification of PLD4 levels was performed using the ImageJ software.

### Sandwich enzyme-linked immunosorbent assay

EVs present in 1000 μg of human plasma samples, centrifuged at 2000 × *g* for 10 min and subsequently at 10 000 × *g* for 30 min, were isolated using the CD63-Capture Human Exosome enzyme-linked immunosorbent assay (ELISA) Kit (FUJIFILM Wako, Osaka, Japan). All procedures strictly adhered to the manufacturer’s instructions. A biotinylated anti-human PLD4 antibody, diluted in antibody reaction buffer (1/100), was added to each well and incubated for 1 h. Thereafter, each well was washed thrice with 320 μL of washing buffer. Streptavidin-horseradish peroxidase (HRP) from the CD63-Capture Human Exosome ELISA Kit was diluted with antibody reaction buffer (1/100) and added to each well and incubated for 2 h. After five washes with 320 μL of washing buffer, 3,3’,5,5’-Tetramethylbenzidine (TMB) solution was added to each well and incubated for 30 min. The reaction was then halted by adding stop solution to each well, followed by the measurement of absorbance at 450 nm and 620 nm [27].

### Nanoparticle tracking analysis

Particle number and size distribution within the plasma samples were determined by nanoparticle tracking analysis (NTA) using a NanoSight NS300 (Malvern Panalytical, Malvern, UK) configured with a 405 nm laser and a high-sensitivity scientific CMOS camera [27–29]. The samples were diluted (plasma, 10:590) in filtered PBS and then analyzed at 24° C. Videos of 15 × 60 s duration were captured with a camera level of 15. The acquired data were analyzed using the NTA 3.2 Dev Build 3.2.16 software with a detection threshold set at 8 [21].

### Statistical analysis

Student’s t-tests were used to compare the intervention and control groups. Statistical significance was set at P < 0.05 [20].

## Results

### Plasma PLD4 is predominantly associated with extracellular vesicles

PLD4 contains a transmembrane domain and is primarily localized in endosomes and lysosomes. Given that endosomes form microvesicular bodies and are a major source of extracellular vesicles, we hypothesized that PLD4 may be released into plasma through its association with extracellular vesicles.

**Fig 1:**
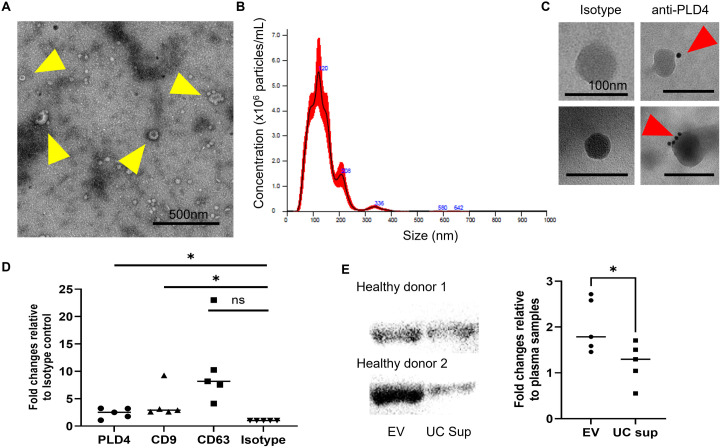
PLD4-containing EVs circulate in human healthy control plasma. (A) Ultrastructural features of extracellular vesicles (EVs) in healthy human plasma. Yellow arrowheads indicate extracellular vesicles. Scale bar; 500 nm (B) Nanoparticle tracking analysis depicting the size of purified EVs from healthy controls. (C) Immunoelectron microscopy revealing anti-PLD4 immunostaining of purified EVs, as opposed to the isotype control. Red arrow heads indicate PLD4 labeled with 10 nm gold. Scale bar; 100 nm. (D) Quantification of PLD4, CD63, and CD9 on CD63-positive EVs using ELISA, relative to the isotype control IgG (*n* = 5). (E) Western blot analysis confirming the presence of PLD4 in purified EVs, rather than the supernatant of ultracentrifuged plasma (UC sup) as EV-free fraction. The histogram indicates higher PLD4 expression levels in purified EVs than in UC sup (*n* = 5). *P < 0.05.

To test this hypothesis, we first isolated extracellular vesicles (EVs), including exosomes and microvesicles, from human plasma using ultracentrifugation. Upon confirming the presence of exosomes, approximately 100 nm in size, through transmission electron microscopy (TEM) and nanoparticle tracking analysis (NTA) (Figs 1A-B), we proceeded to assess the presence of PLD4 using immunoelectron microscopy (IEM). As a result, colloidal-gold-conjugated streptavidin was observed on the outer surface of the EV membrane upon application of the biotinylated anti-human PLD4 antibody, whereas it was not detected with the control antibody (Fig 1C). To further confirm the co-presence of purified human plasma derived-EVs and PLD4, we captured EVs on a plate immobilized with anti-CD63 antibodies, which bind to the EV membrane, and evaluated the presence of PLD4 using ELISA (Fig 1D). The results confirmed that PLD4 is indeed co-present on EVs expressing CD63.

PLD3, which shares high amino acid sequence homology and functional similarity with PLD4, has been reported to be cleaved at its transmembrane region by cathepsin, resulting in the release of a soluble form into lysosomes [30]. To investigate the possibility that PLD4, like PLD3, exists as a soluble form in plasma, lacking its transmembrane domain, we evaluated the presence of PLD4 in both EVs purified by ultracentrifugation and the post-purification supernatant via Western blotting (Fig 1E). While the presence of PLD4 in a soluble form—lacking the hydrophobic regions and therefore dissolved in plasma—could not be entirely ruled out, the results indicate that PLD4 predominantly exists in human plasma in association with EVs.

### B cell activation promotes PLD4 translocation from the trans-Golgi network to multivesicular bodies

**Fig 2:**
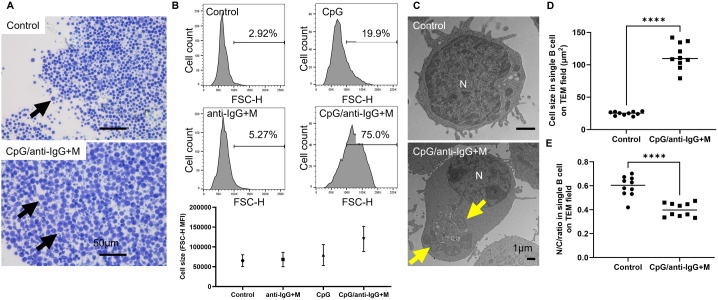
CpG/anti-IgG + M stimulation induces cellular hypertrophy. (A) Toluidine blue staining of purified B cells. Black arrows indicate activated B cells. (B) Flow cytometry identified highly purified B cells isolated using the EasySep^TM^ Pan-B cell Enrichment Kit, according to the manufacture’s protocol. The histogram exhibits B cell size enlargement induced by CpG/anti-IgG + M stimulation. N: nucleus. (C) Transmission electron microscopy (TEM) shows increased cytoplasm and mitochondrial proliferation following CpG/anti-IgG + M stimulation. Yellow arrows indicate mitochondria. (D,E) Histograms indicate the increase in cell size and the decrease in the nuclear-to-cytoplasmic (N/C) ratio following CpG/anti-IgG + M stimulation.

**Fig 3:**
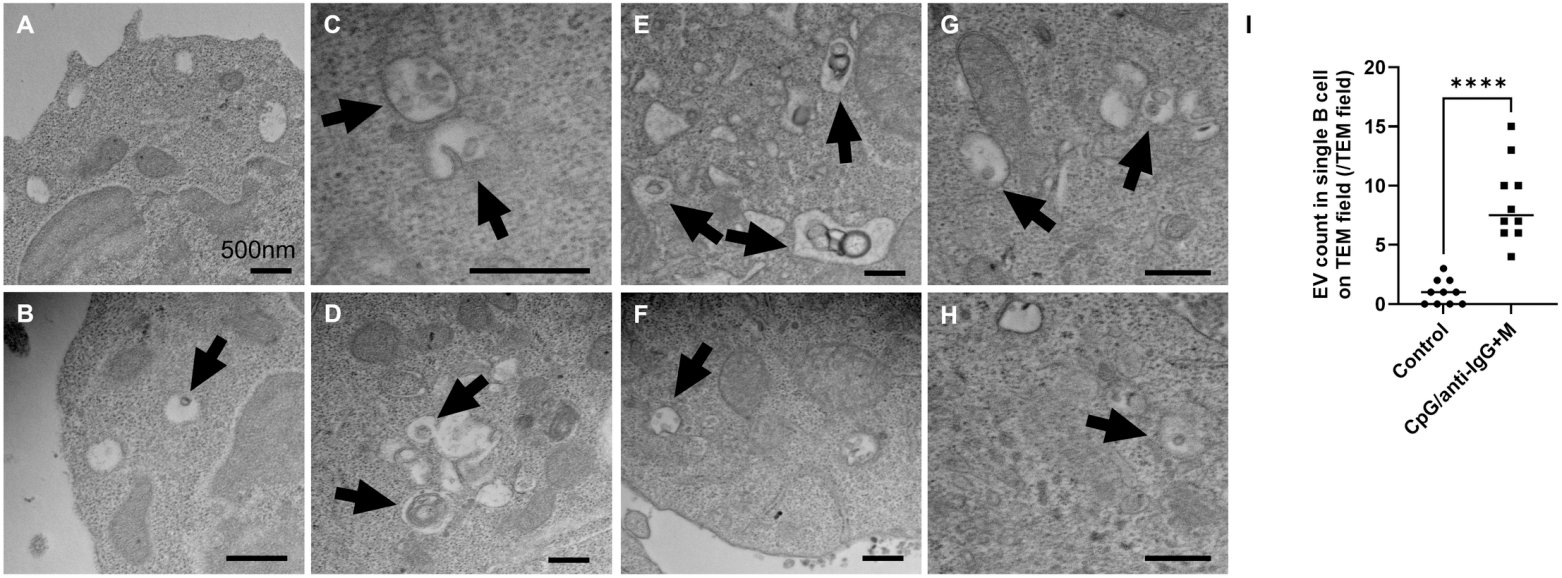
CpG/anti-IgG + M stimulation enhances the accumulation of ILVs. (A,B) Transmission electron microscopic observation of control B cells. (C-H) Transmission electron microscopic observation of CpG/anti-IgG + M BCR- and TLR9--stimulated B cells. Black arrows indicate multivesicular bodies (MVBs). (I) Histogram showing a significant increase in the number of intraluminal vesicles in CpG/anti-IgG + M stimulated B cells. Scale bar, 500 nm. *P < 0.001.

Next, we investigated the formation of EVs linked to PLD4 in vitro using human primary cells. According to publicly available human transcriptome data, this phospholipase is preferentially expressed in the immune system, notably in plasmacytoid dendritic cells, B cells, and monocyte/macrophage lineages, while its expression is undetectable in granulocytes and T cell lineages. Moreover, previous reports indicate that EVs originating from hematopoietic cells in human plasma are predominantly derived from B cells, following platelets [31]. Given this background, we conducted in vitro experiments focusing on B cells, as these can be isolated in sufficient quantities from healthy human blood sample to assess in vitro EV production. Human B cells have been documented that they release EVs upon activation by signals such as B cell receptor (BCR) engagement [32]. In our study, the combination of a TLR9 agonist and BCR signaling resulted in the most significant increase in both cell size and nuclear-to-cytoplasmic ratio. (Fig 2). Moreover, TEM revealed an increase in the number of intraluminal vesicles (ILVs) within multivesicular bodies (MVBs) following BCR- and TLR9- stimulation with CpG/anti-IgG + M (Fig 3). Notably, endosomes containing these MVBs were in close proximity to mitochondria, suggesting that mitochondria may serve as a biological energy source for MVB formation following activation stimuli (Figs 2B, 3G).

### Activated B cells are a resource of PLD4-containing extracellular vesicles in human plasma

**Fig 4:**
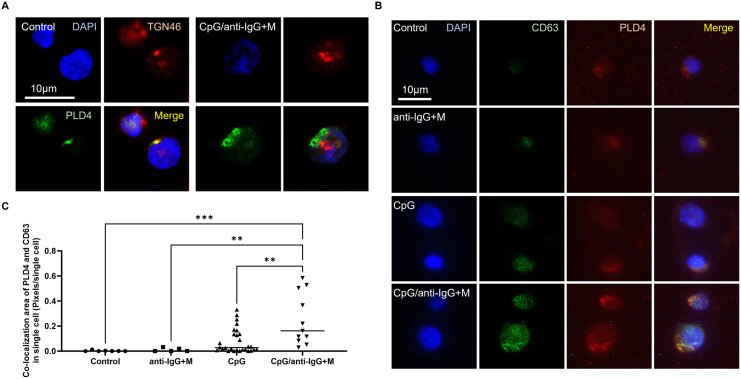
CpG/anti-IgG + M stimulation enhances PLD4 localization to MVBs. (A) Immunofluorescence microscopy displaying localization of PLD4 (red) at TGN46-tagged trans Golgi networks (green) in resting B cells and translocation of PLD4 from trans Golgi networks in CpG/anti-IgG + M stimulated B cells, compared to the control cells. (B) Immunofluorescence microscopy shows enhanced expression of PLD4 (red) and co-localization of PLD4 and CD63 (green) in CpG/anti-IgG + M stimulated B cells, compared to the control cells. Cell membranes stained with MemBrite appeared thicker in CpG/anti-IgG + M stimulated B cells than in the control cells. (C) Statistical analysis revealing enhanced PLD4 expression in each CpG/anti-IgG + M stimulated B cell compared to the control cells. Bar; 10 μm, **P < 0.01, ***P < 0.001.

**Fig 5:**
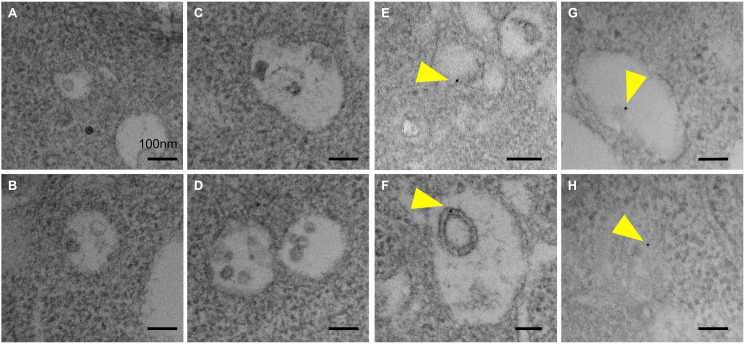
CpG/anti-IgG + M stimulation enhances PLD4-containing MVBs in B cell cytoplasm. Immunogold labeling for PLD4. (A-D) Control B cells; (E-H) CpG/anti-IgG + M stimulated B cells. Yellow arrowheads indicate the presence of PLD4 on intraluminal vesicles (ILVs). Scale bar, 100 nm.

**Fig 6:**
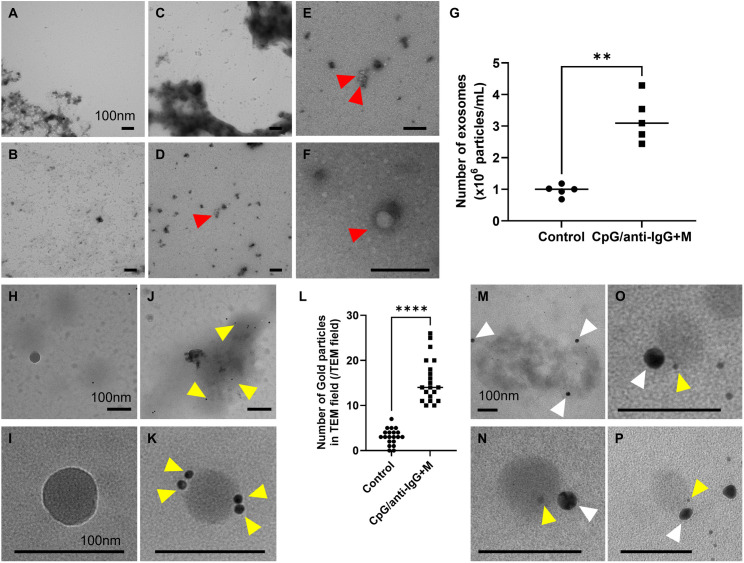
CpG/anti-IgG + M stimulation enhances PLD4-containing EV secretion. (A-F) Transmission electron microscopy (TEM) indicates as increased secretion of extracellular vesicles (EVs) solely in CpG/anti-IgG + M BCR- and TLR9--stimulated B cells compared to the control cells. (A) Control cells; (B) anti-IgG + M mono-stimulated B cells; (C) CpG mono-stimulated B cells; (D-F) CpG/anti-IgG + M stimulated B cells. Red arrowheads indicate EVs. (G) Nanoparticle tracking analysis revealed an increased number of EVs in CpG/anti-IgG + M stimulated B cells, compared to the control cells. (H-K) Immunoelectron microscopic study revealed an increased presence of PLD4-positive EVs in CpG/anti-IgG + M stimulated B cells, compared to the control cells. (H,I) Control cells; (J,K) CpG/anti-IgG + M stimulated B cells. Yellow arrowheads indicate PLD4 tagged by gold particles. (L) Statistical analysis counting PLD4-tagged gold particles in 10 randomly selected TEM fields. Scale bar, 100 nm. *P < 0.001. (M-P) Immunoelectron microscopic study revealed anti-PLD4 immunostaining of CD19-tagged plasma EVs. Yellow arrowheads indicate PLD4 tagged by 5nm gold particles, while white arrow heads show CD19 tagged by 20nm gold particles. Scale bar; 100 nm.

Under the aforementioned conditions, the intracellular localization of PLD4 in B cells stimulated in vitro was analyzed using fluorescence microscopy. In unstimulated control B cells, the PLD4 protein was primarily localized in the Golgi apparatus, whereas, in stimulated cells, it was observed to shift to CD63-positive late endosomes (Figs 4A-C). Additionally, IEM analysis confirmed the relocation of PLD4 in activated B cells, demonstrating that it was bound to MVB membranes within the endosomes induced by activation (Fig 5). Indeed, no accumulation of PLD4 in MVB was detected in HEK293T cells, which reported not to express PLD4 [33]. However, accumulation of PLD4 was detected in PLD4-overexpressing HEK293T cells (S1 Fig). Furthermore, ultracentrifugation of the culture supernatant from these stimulated B cells, followed by immunoelectron microscopy (IEM), confirmed the presence of PLD4 on the membrane of the EVs released by these cells (Figs 6A-L). Lastly, to confirm the origin of EVs carrying PLD4 in human plasma, co-staining of EVs isolated from this source using lineage markers indicated that many of the vesicles binding the enzyme also expressed CD19 (Figs 6M-P). While these findings do not entirely rule out the involvement of other cell types, they strongly suggest that activated B cells are a key source of PLD4-bearing EVs in human plasma.

## Discussion

Our study shows that PLD4 is present in human plasma and predominantly associated with extracellular vesicles (EVs) secreted by activated B cells. Upon stimulation, PLD4 translocates from the Golgi apparatus to CD63-positive late endosomes, aiding its incorporation into intraluminal vesicles (ILVs), which are subsequently released as exosomes into the extracellular space.

This study provides clear evidence that PLD4 circulates in the human bloodstream as a membrane-associated component of EVs. While publicly available database indicated the presence of PLD4 in plasma, our findings specifically demonstrate its extracellular localization in association with EVs. The potential for PLD4 cleavage and solubilization remains an open question, as suggested by studies on PLD3, a closely related family member. Western blot analysis detected PLD4 in both EVs and the post-ultracentrifugation supernatant, though the limited purity of isolated fractions precludes definitive conclusions regarding the predominant form of PLD4 in plasma.

Mechanistically, our study uncovers that CpG and anti-IgM/IgG stimulation of B cells promotes the translocation of PLD4 from the trans-Golgi network (TGN) to the plasma membrane and multivesicular bodies (MVBs). This dynamic relocation correlates with ILV formation, particularly in CpG/anti-IgM + IgG-stimulated cells, whereas PLD4 remains localized in the TGN in unstimulated cells. Notably, TLR9 stimulation alone enhances PLD4 recruitment to the plasma membrane and promotes ILV formation, as evidenced by increased co-localization with CD63, while BCR stimulation alone does not induce significant changes in cell size or CD63 expression. These findings suggest that TLR9 signaling plays a dominant role in PLD4 trafficking to EVs.

Furthermore, transmission electron microscopy (TEM) revealed frequent spatial proximity between mitochondria and PLD4-containing ILVs, although mitochondria were not directly incorporated into the vesicles. This observation raises important questions about the potential involvement of mitochondria in EV biogenesis and secretion, warranting further investigation.

In summary, our findings outline a mechanistic pathway by which PLD4 is trafficked to EVs in response to specific B cell activation signals. By providing direct evidence of PLD4’s extracellular localization in EVs, this study deepens our knowledge of its potential roles in intercellular communication and immune regulation. Future studies should investigate the physiological and pathological implications of PLD4-containing EVs in immune homeostasis and disease progression.

## Supporting information

S1 File Supporting information. (XLSX)

S1 Fig Validation of specificity of anti-PLD4 antibody in immunoelectron microscopy. Immunogold labeling for PLD4. (A-C) HEK293T cells; (D-F) PLD4-overexpressing HEK293T cells. The framed area in A is magnified in B, and that in D is still magnified in E. Yellow arrow heads (10 nm-gold labeled PLD4). N: nucleus; G: Golgi’s apparatus.
(ZIP)

## References

[1] Santa P, Garreau A, Serpas L, Ferriere A, Blanco P, Soni C, et al. The Role of Nucleases and Nucleic Acid Editing Enzymes in the Regulation of Self-Nucleic Acid Sensing. Front Immunol. 2021;12:629922. doi: 10.3389/fimmu.2021.629922 33717156 PMC7952454

[2] Gavin AL, Huang D, Blane TR, Thinnes TC, Murakami Y, Fukui R, et al. Cleavage of DNA and RNA by PLD3 and PLD4 limits autoinflammatory triggering by multiple sensors. Nat Commun. 2021;12(1):5874. doi: 10.1038/s41467-021-26150-w 34620855 PMC8497607

[3] Egea-Jimenez AL, Zimmermann P. Phospholipase D and phosphatidic acid in the biogenesis and cargo loading of extracellular vesicles. J Lipid Res. 2018;59(9):1554–60. doi: 10.1194/jlr.R083964 29853529 PMC6121939

[4] Gavin AL, Huang D, Huber C, Mårtensson A, Tardif V, Skog PD, et al. PLD3 and PLD4 are single-stranded acid exonucleases that regulate endosomal nucleic-acid sensing. Nat Immunol. 2018;19(9):942–53. doi: 10.1038/s41590-018-0179-y 30111894 PMC6105523

[5] Otani Y, Yamaguchi Y, Sato Y, Furuichi T, Ikenaka K, Kitani H, et al. PLD$ is involved in phagocytosis of microglia: expression and localization changes of PLD4 are correlated with activation state of microglia. PLoS One. 2011;6(11):e27544. doi: 10.1371/journal.pone.0027544 22102906 PMC3216956

[6] Trivedi P, Kumar RK, Iyer A, Boswell S, Gerarduzzi C, Dadhania VP, et al. Targeting Phospholipase D4 Attenuates Kidney Fibrosis. J Am Soc Nephrol. 2017;28(12):3579–89. doi: 10.1681/ASN.2016111222 28814511 PMC5698063

[7] Chen W-C, Wang W-C, Okada Y, Chang W-P, Chou Y-H, Chang H-H, et al. rs2841277 (PLD4) is associated with susceptibility and rs4672495 is associated with disease activity in rheumatoid arthritis. Oncotarget. 2017;8(38):64180–90. doi: 10.18632/oncotarget.19419 28969061 PMC5609993

[8] Akizuki S, Ishigaki K, Kochi Y, Law S-M, Matsuo K, Ohmura K, et al. PLD4 is a genetic determinant to systemic lupus erythematosus and involved in murine autoimmune phenotypes. Ann Rheum Dis. 2019;78(4):509–18. doi: 10.1136/annrheumdis-2018-214116 30679154

[9] Terao C, Ohmura K, Kawaguchi Y, Nishimoto T, Kawasaki A, Takehara K, et al. PLD4 as a novel susceptibility gene for systemic sclerosis in a Japanese population. Arthritis Rheum. 2013;65(2):472–80. doi: 10.1002/art.37777 23124809

[10] Gavin AL, Blane TR, Thinnes TC, Gerlt E, Marshak-Rothstein A, Huang D, et al. Disease in the Pld4thss/thss Model of Murine Lupus Requires TLR9. Immunohorizons. 2023;7(8):577–86. doi: 10.4049/immunohorizons.2300058 37555846 PMC10441812

[11] Shi G, Abbott KN, Wu W, Salter RD, Keyel PA. Dnase1L3 Regulates Inflammasome-Dependent Cytokine Secretion. Front Immunol. 2017;8:522. doi: 10.3389/fimmu.2017.00522 28533778 PMC5420570

[12] Sisirak V, Sally B, D’Agati V, Martinez-Ortiz W, Özçakar ZB, David J, et al. Digestion of Chromatin in Apoptotic Cell Microparticles Prevents Autoimmunity. Cell. 2016;166(1):88–101. doi: 10.1016/j.cell.2016.05.034 27293190 PMC5030815

[13] Belot A, Rice GI, Omarjee SO, Rouchon Q, Smith EMD, Moreews M, et al. Contribution of rare and predicted pathogenic gene variants to childhood-onset lupus: a large, genetic panel analysis of British and French cohorts. Lancet Rheumatol. 2020;2(2):e99–109. doi: 10.1016/S2665-9913(19)30142-0 38263665

[14] Acosta-Herrera M, Kerick M, González-Serna D, Myositis Genetics Consortium, Scleroderma Genetics Consortium, Wijmenga C, et al. Genome-wide meta-analysis reveals shared new loci in systemic seropositive rheumatic diseases. Ann Rheum Dis. 2019;78(3):311–9. doi: 10.1136/annrheumdis-2018-214127 30573655 PMC6800208

[15] Uhlén M, Fagerberg L, Hallström BM, Lindskog C, Oksvold P, Mardinoglu A, et al. Proteomics. Tissue-based map of the human proteome. Science. 2015;347(6220):1260419. doi: 10.1126/science.1260419 25613900

[16] Human Protein Atlas [Internet]. [cited 2024 Aug 04] Available from: https://www.proteinatlas.org

[17] Rincón-Arévalo H, Burbano C, Atehortúa L, Rojas M, Vanegas-García A, Vásquez G, et al. Modulation of B cell activation by extracellular vesicles and potential alteration of this pathway in patients with rheumatoid arthritis. Arthritis Res Ther. 2022;24(1):169. doi: 10.1186/s13075-022-02837-3 35842663 PMC9287863

[18] Oved K, Ziv O, Jacob-Hirsch J, Noy R, Novak H, Makler O, et al. A novel postpriming regulatory check point of effector/memory T cells dictated through antigen density threshold-dependent anergy. J Immunol. 2007;178(4):2307–17. doi: 10.4049/jimmunol.178.4.2307 17277136

[19] Shankar S, Stolp J, Juvet SC, Beckett J, Macklin PS, Issa F, et al. Ex vivo-expanded human CD19+TIM-1+ regulatory B cells suppress immune responses in vivo and are dependent upon the TIM-1/STAT3 axis. Nat Commun. 2022;13(1):3121. doi: 10.1038/s41467-022-30613-z 35660734 PMC9166804

[20] Betsuyaku T, Eid N, Ito Y, Tanaka Y, Otsuki Y, Kondo Y. Ethanol enhances thymocyte apoptosis and autophagy in macrophages of rat thymi. Histol Histopathol. 2017;32(9):963–75. doi: 10.14670/HH-11-861 28026004

[21] Hasan UA, Zannetti C, Parroche P, Goutagny N, Malfroy M, Roblot G, et al. The human papillomavirus type 16 E7 oncoprotein induces a transcriptional repressor complex on the Toll-like receptor 9 promoter. J Exp Med. 2013;210(7):1369–87. doi: 10.1084/jem.20122394 23752229 PMC3698525

[22] Théry C, Witwer KW, Aikawa E, Alcaraz MJ, Anderson JD, Andriantsitohaina R, et al. Minimal information for studies of extracellular vesicles 2018 (MISEV2018): a position statement of the International Society for Extracellular Vesicles and update of the MISEV2014 guidelines. J Extracell Vesicles. 2018;7(1):1535750. doi: 10.1080/20013078.2018.1535750 30637094 PMC6322352

[23] Ito Y, Taniguchi K, Kuranaga Y, Eid N, Inomata Y, Lee S-W, et al. Uptake of MicroRNAs from Exosome-Like Nanovesicles of Edible Plant Juice by Rat Enterocytes. Int J Mol Sci. 2021;22(7):3749. doi: 10.3390/ijms22073749 33916868 PMC8038500

[24] Lucocq JM, Gawden-Bone C. Quantitative assessment of specificity in immunoelectron microscopy. J Histochem Cytochem. 2010;58(10):917–27. doi: 10.1369/jhc.2010.956243 20458060 PMC2942744

[25] Yokota S. Historical survey on chromatoid body research. Acta Histochem Cytochem. 2008;41(4):65–82. doi: 10.1267/ahc.08010 18787638 PMC2532602

[26] Smyth CM, Logan G, Boadle R, Rowe PB, Smythe JA, Alexander IE. Differential subcellular localization of CD86 in human PBMC-derived macrophages and DCs, and ultrastructural characterization by immuno-electron microscopy. Int Immunol. 2005;17(2):123–32. doi: 10.1093/intimm/dxh193 15623548

[27] Nakai W, Yoshida T, Diez D, Miyatake Y, Nishibu T, Imawaka N, et al. A novel affinity-based method for the isolation of highly purified extracellular vesicles. Sci Rep. 2016;6:33935. doi: 10.1038/srep33935 27659060 PMC5034288

[28] Kulka M, Brennan K, Mc Gee M. Investigation of canine extracellular vesicles in diffuse large B-cell lymphomas. PLoS One. 2022;17(9):e0274261. doi: 10.1371/journal.pone.0274261 36125986 PMC9488776

[29] Walker SA, Davidovich I, Yang Y, Lai A, Goncalves JP, Deliwala V, et al. Sucrose-based cryoprotective storage of extracellular vesicles. Extracell Vesicle. 2022;1:100016. doi: 10.1016/j.vesic.2022.100016 38665624 PMC11044822

[30] Gonzalez AC, Schweizer M, Jagdmann S, Bernreuther C, Reinheckel T, Saftig P, et al. Unconventional Trafficking of Mammalian Phospholipase D3 to Lysosomes. Cell Rep. 2018;22(4):1040–53. doi: 10.1016/j.celrep.2017.12.100 29386126

[31] Li Y, He X, Li Q, Lai H, Zhang H, Hu Z, et al. EV-origin: Enumerating the tissue-cellular origin of circulating extracellular vesicles using exLR profile. Comput Struct Biotechnol J. 2020;18:2851–9. doi: 10.1016/j.csbj.2020.10.002 33133426 PMC7588739

[32] Phan H-D, Longjohn MN, Gormley DJB, Smith RH, Dang-Lawson M, Matsuuchi L, et al. CD24 and IgM Stimulation of B Cells Triggers Transfer of Functional B Cell Receptor to B Cell Recipients Via Extracellular Vesicles. J Immunol. 2021;207(12):3004–15. doi: 10.4049/jimmunol.2100025 34772696

[33] Singh S, Dransfeld UE, Ambaw YA, Lopez-Scarim J, Farese RV Jr, Walther TC. PLD3 and PLD4 synthesize S,S-BMP, a key phospholipid enabling lipid degradation in lysosomes. Cell. 2024;187(24):6820–34.e24. doi: 10.1016/j.cell.2024.09.036 39423811 PMC12055030

