# Correction: Activated human B cells produce phospholipase D4-containing extracellular vesicles

**DOI:** 10.1371/journal.pone.0337864

**Published:** 2025-12-01

**Authors:** The PLOS One Staff

## Notice of republication

This article was republished on November 15, 2025, to correct an error in the Supporting Information files. A corrupted version of S1 Fig was published in error. The publisher apologises for this error. Please download this article again to view the correct version.

## Supporting information

S1 File Originally published, uncorrected article. (PDF)

S2 File Republished, corrected article. (PDF)

S1 Fig Validation of specificity of anti-PLD4 antibody in immunoelectron microscopy. Immunogold labeling for PLD4. (A-C) HEK293T cells; (D-F) PLD4-overexpressing HEK293T cells. The framed area in A is magnified in B, and that in D is still magnified in E. Yellow arrow heads (10 nm-gold labeled PLD4). N: nucleus; G: Golgi’s apparatus.
(TIF)
