# Supplementary figures and images for: Activated human B cells produce phospholipase D4-containing extracellular vesicles
Source: PLoS One. 2025 Aug 14;20(8):e0329832. doi: 10.1371/journal.pone.0329832 (PMC12352752; doi:10.1371/journal.pone.0329832)

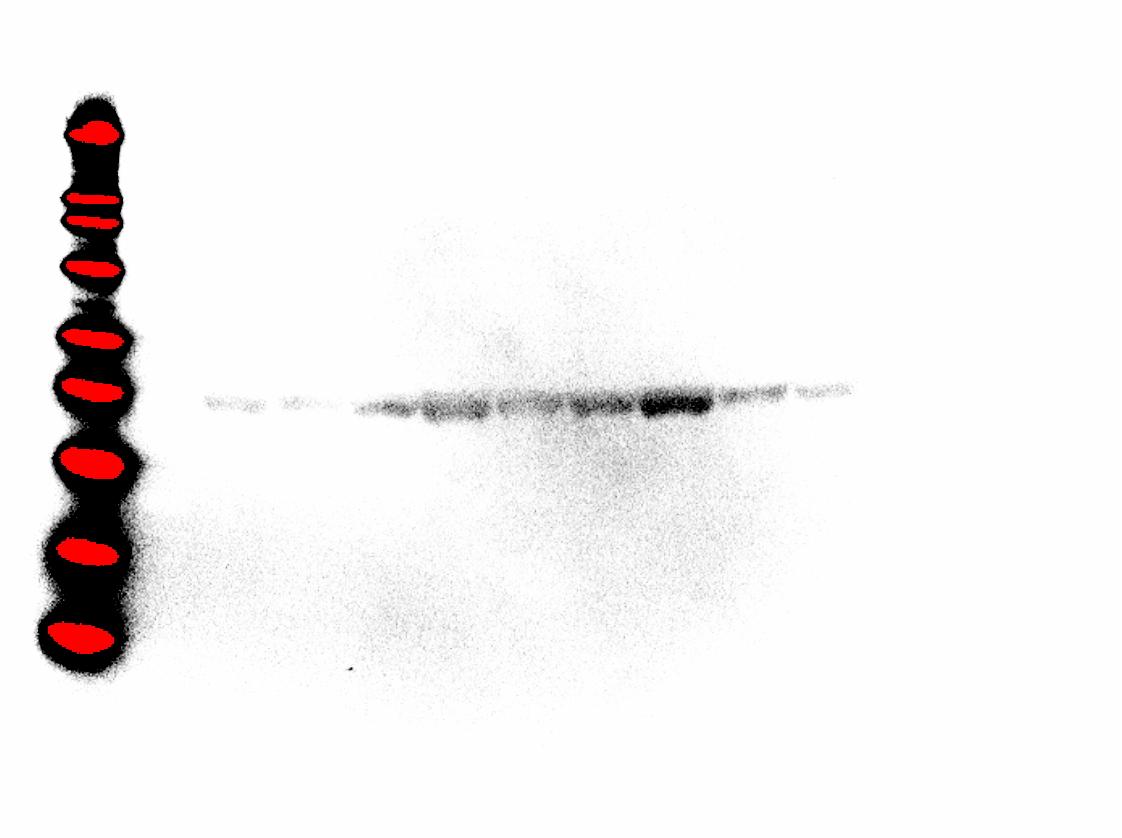

Supplement: S1 Fig — Immunogold labeling for PLD4. (A-C) HEK293T cells; (D-F) PLD4-overexpressing HEK293T cells. The framed area in A is magnified in B, and that in D is still magnified in E. Yellow arrow heads (10 nm-gold labeled PLD4). N: nucleus; G: Golgi’s apparatus. (ZIP) [file pone.0329832.s002.zip › Raw image Figure 1E.tif]

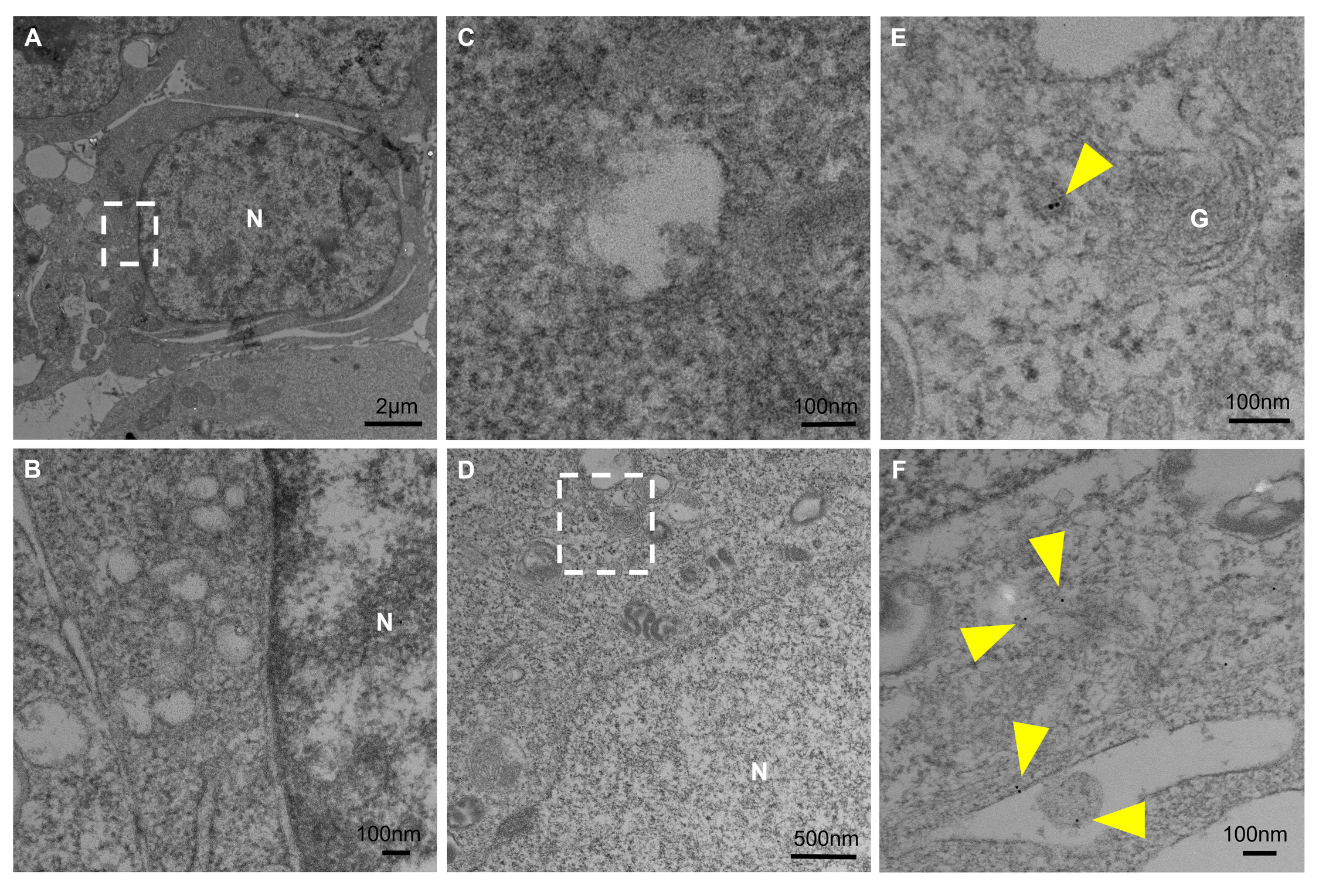

Supplement: S1 Fig — Immunogold labeling for PLD4. (A-C) HEK293T cells; (D-F) PLD4-overexpressing HEK293T cells. The framed area in A is magnified in B, and that in D is still magnified in E. Yellow arrow heads (10 nm-gold labeled PLD4). N: nucleus; G: Golgi’s apparatus. (ZIP) [file pone.0329832.s002.zip › Supplementary figure 1.tif]
