# Supplementary figures and images for: Correction: Activated human B cells produce phospholipase D4-containing extracellular vesicles
Source: PLoS One. 2025 Dec 1;20(12):e0337864. doi: 10.1371/journal.pone.0337864 (PMC12668510; doi:10.1371/journal.pone.0337864)

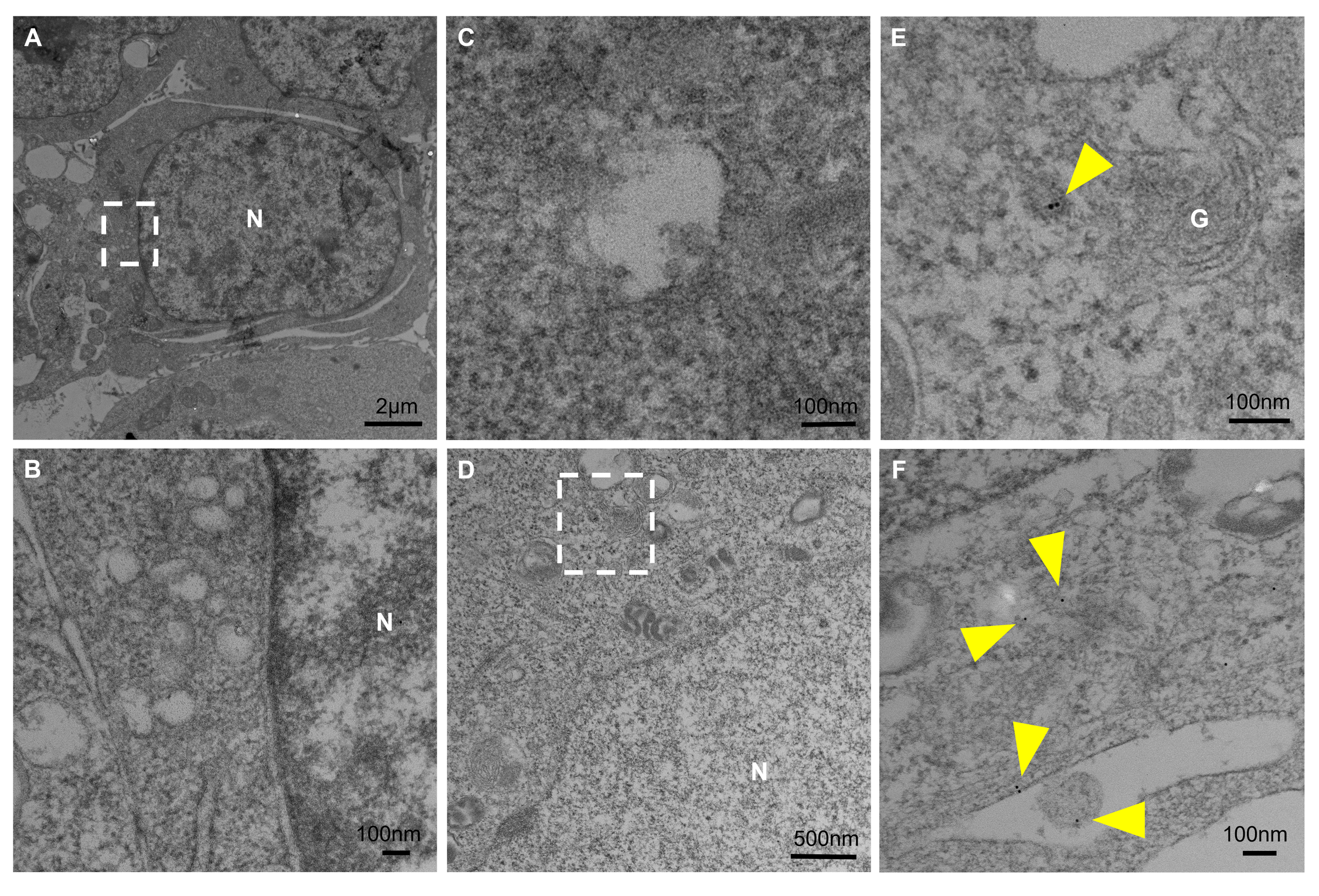

Supplement: S1 Fig — Immunogold labeling for PLD4. (A-C) HEK293T cells; (D-F) PLD4-overexpressing HEK293T cells. The framed area in A is magnified in B, and that in D is still magnified in E. Yellow arrow heads (10 nm-gold labeled PLD4). N: nucleus; G: Golgi’s apparatus. (TIF) [file pone.0337864.s003.tif]
